# Ferroptosis research based on bibliometric and visual analysis: mechanism exploration and clinical application prospects in gastric cancer, prostate cancer, leukemia, and brain tumors

**DOI:** 10.3389/fmed.2025.1640497

**Published:** 2025-10-24

**Authors:** Mingkun Yu, Wenze Cui, Yinghua Hu, Wenhuan Song, Zujun Wang, Wenyu Chen, Xiaolu Ji, Chen Tang, Jiantao Lv, Tao Hao

**Affiliations:** ^1^Shandong University of Traditional Chinese Medicine, Jinan, China; ^2^Binzhou Medical College Affiliated Traditional Chinese Medicine Hospital, Binzhou, China; ^3^Binzhou Medical University, Yantai, Shandong, China; ^4^Binzhou People’s Hospital, Binzhou, Shandong, China; ^5^Binzhou Medical University Hospital, Binzhou, Shandong, China

**Keywords:** ferroptosis, bibliometric analysis, gastric cancer, prostate cancer, leukemia, brain tumors, therapeutic resistance

## Abstract

**Purpose:**

Ferroptosis, iron-dependent cell death, holds significant therapeutic potential in oncology. This study aimed to map global research trends (2014–2024) of ferroptosis in gastric cancer, prostate cancer, leukemia, and brain tumors via bibliometric analysis, identifying key developments and clinical prospects.

**Methods:**

Publications from Web of Science, Scopus, and PubMed were analyzed using VOSviewer, CiteSpace for output, collaborations, influential authors/works (co-citation), and keyword trends (burst detection).

**Results:**

Research output surged, led by China and the USA. Foundational authors (Dixon, Yang, Stockwell) were highly co-cited. Emerging hotspots include overcoming therapy resistance, tumor microenvironment modulation, immunotherapy integration, and nanotechnology applications. Cancer-specific foci like GPx4 (gastric) and p53 (prostate) pathways were evident.

**Conclusion:**

This analysis provides a comprehensive map of the evolving ferroptosis research landscape across these four cancers, revealing a distinct shift towards translational applications. The findings of this study provide a valuable framework for guiding future research and for the prioritization of clinical strategies targeting ferroptosis.

## 1 Background

In contrast to conventional cell death modalities such as apoptosis and necrosis, ferroptosis is a distinct form of regulated programmed cell death characterized by its tight regulatory control. Driven by iron-catalyzed reactions, ferroptosis unfolds through a complex signaling network, with the extensive peroxidation of lipids serving as its central execution step (1). Ferroptosis has garnered intense interest within the oncology community, driven by its distinct mechanistic properties and its promise as a novel therapeutic strategy. It stands tall in cancer therapy. It is not merely a new-found cell-death route. The induction of ferroptosis has been demonstrated to overcome therapeutic resistance to conventional anticancer agents. Targeting ferroptosis offers a novel therapeutic strategy for the treatment of refractory or recurrent tumors (2).

A diverse range of malignancies, including gastric cancer, prostate cancer, leukemia, and brain tumors, impose a substantial burden on global healthcare systems. The intricate role of ferroptosis in pathophysiology is a unifying feature across these clinically distinct malignancies, each characterized by its own biological specificities. Each of these cancers, occurring worldwide, possesses its own set of distinct biological characteristics. Ferroptosis plays an intricate and pivotal role in the pathophysiology of these malignancies (3–6). Ferroptosis has been implicated in key aspects of gastric cancer pathophysiology, including tumor progression, metastasis, and therapeutic response. Distinct molecular signatures in metastatic gastric adenocarcinoma (GAC) are now being elucidated through sophisticated techniques, including single-cell transcriptomics and immunoassays. Cancer cells dodge ferroptosis. Malignant cells often upregulate the expression of glutathione peroxidase 4 (GPX4) during tumor progression (7). Recent reviews have highlighted that ferroptosis induction, as a potential anti-cancer strategy, may overcome therapeutic resistance in leukemia (8–13). Although studies on ferroptosis in prostate cancer are still limited, the fundamental principles governing this process, particularly its regulation through pathways like the tumor protein p53 (p53) pathway and lipid peroxidation, suggest its potential as a promising therapeutic strategy for this malignancy (14–16). The treatment of brain tumors remains a formidable challenge due to the restrictive nature of the blood–brain barrier (BBB), which severely limits the delivery of therapeutic agents to the central nervous system (CNS). Recent studies have identified ferroptosis as a potential mechanism to mitigate BBB dysfunction and improve the efficacy of brain tumor therapies (17). The rapidly expanding body of research on ferroptosis and its implications in cancer has led to a surge in related publications (18–20). This increase in literature has created significant challenges for researchers seeking systematic summaries and comprehensive evaluations. This bibliometric analysis provides a robust framework for identifying high-impact research, leading institutions, core journals, and key collaboration networks, while also highlighting emerging research hotspots and trends in gastric cancer, prostate cancer, leukemia, and brain tumors. This paper dives deep into Ferroptosis within cancer research. It lays out clear-cut insights for future work. It focuses on crafting new-wave therapeutic plans. It also aims to boost cancer treatment success.

## 2 Methods

### 2.1 Data collection

This paper zeroes in on four cancer types. It sifts through relevant studies from Web of Science, Scopus, PubMed databases. These span 2014 to 2024. The last search was conducted on January 12, 2025. The cancers include gastric cancer, prostate cancer, leukemia, brain tumors. The search pulls in many ferroptosis-related terms. These cover “ferroptosis,” “iron level,” “iron status,” “serum iron,” “iron concentration,” “iron metabolism,” “iron homeostasis.” This ensures wide-ranging data grab. Each cancer type gets its own search tweaks. Custom-fit terms shape these methods. Gastric cancer’s search terms were “gastric cancer,” “gastric carcinoma,” “cancer of the stomach,” “gastric tumor,” and “stomach neoplasm.” “Prostate cancer,” “cancer of the prostate gland,” “prostatic cancer,” and “prostatic neoplasms” were among the terms for prostate cancer. Terms like “leukemia,” “leucocythemia,” and “leukemias” were used to search for leukemia. “Brain tumor” and “cerebral tumor” among many other brain tumor-related phrases were used. Iron death-related phrases were combined with cancer terms using the Boolean operator AND, hence guaranteeing the accuracy of the dataset for each cancer type. Journal articles, reviews, and conference papers were included in the retrieval scope.

### 2.2 Bibliometric indicators

Using four main bibliometric tools, this paper assesses cancer-related research: publication volume, citation frequency, impact factor (IF), and keyword burst detection.

Publication volume is a measure of the total number of publications published on each cancer type, hence indicating the degree and intensity of research activity in a certain field. Usually, a larger publication volume indicates a more active and widely investigated field of study.

Citation frequency tracks how often individual articles are referenced in later studies, hence measuring their academic influence. Usually, a larger citation count shows more academic impact and more awareness inside the research community.

Impact factor (IF) is the mean citation count for papers published in a particular publication. This measure reveals the renown of the journal and the academic importance of the work it publishes. Studies released in high-impact journals are often seen as more powerful and authoritative.

Keyword burst detection is a bibliometric method for identifying emerging research frontiers by analyzing sharp increases in keyword frequency over a defined period. This method enables researchers to capture the dynamic evolution of the cancer research landscape, identify significant intellectual trends, and highlight potential directions for future investigation. The onset and duration of keyword bursts, as detected by software like CiteSpace and VOSviewer, serve as quantitative indicators of shifts in research focus and periods of intensified scholarly engagement.

### 2.3 Burst detection

This version is a direct upgrade of the original sentence, replacing informal words with their academic equivalents. It is the most versatile and widely applicable version. Through bibliometric analysis, researchers can track the dynamics of keyword frequency, thereby revealing emergent research themes and evolving intellectual trends. The intensity, onset, and duration of a keyword burst serve to quantify and delineate periods of heightened scholarly focus and concentrated research activity.

### 2.4 Visualization and collaborative network analysis

This paper builds co-citation and keyword co-occurrence networks to investigate the collaborative dynamics across authors, institutions, and nations in cancer research. Examining the visual depiction of these networks helps researchers to find top research teams and nations as well as their patterns of international cooperation and impact. Journal co-citation study also highlights important publications with notable scholarly influence in several cancer research areas, hence stressing their crucial contribution to the development of the discipline.

### 2.5 Data analysis tools

To ensure the precision and uniqueness of the included literature dataset, this study employed a rigorous, multi-level deduplication strategy. The process began with a first-round automated screening using the “Find Duplicates” function in the EndNote literature management software, with “author, year, title” as the combined matching criteria, to efficiently remove the majority of explicit duplicate entries. Recognizing the limitations of automated tools in identifying implicit duplicates caused by subtle discrepancies such as variations in spelling, capitalization, and special characters, we then conducted a second round of manual review: all literature records were sorted alphabetically by “title” for meticulous, one-by-one comparison and merging. To guarantee the absolute purity of the final dataset, a third, supplementary verification was performed by leveraging the uniqueness of the Digital Object Identifier (DOI). To ensure the integrity of the final dataset, a DOI-based deduplication was performed on all sorted records. This rigorous protocol, which integrated the efficiency of automated processing with the precision of manual verification, ensured a robust and reliable dataset for the subsequent bibliometric analysis. The detailed process of screening has been described in detail in the PRISMA flowchart (Supplementary Figure S1).

This study employed specialized software, such as VOSviewer and CiteSpace, to construct and visualize co-citation networks, thereby identifying emergent research frontiers. Analysis of ranked tables and charts, which detail the contributions of leading countries, institutions, and journals, served to identify the field’s most influential research areas. This analysis yields significant implications for shaping the course of future investigations and can inform the strategic direction of research in this field.

In this study, VOSviewer was employed to construct and visualize the bibliographic co-occurrence network, thereby mapping the intellectual structure of the field. The co-occurrence network was constructed using the Full Counting method. To ensure the validity of the analysis, we set minimum thresholds for the units of analysis: a minimum of 5 publications for countries or institutions, and a minimum of 10 occurrences for keywords. Based on this, we employed its built-in Modularity algorithm for cluster analysis to identify core research clusters.

Furthermore, to reveal the temporal evolution and emerging trends of the research field, we conducted a supplementary analysis using CiteSpace software. The specific parameter settings were as follows: Time Slice was set to 1 year, the selection criteria was top *N* = 50, and the Pathfinder algorithm was applied for network pruning to highlight key paths. The burst analysis of keywords was performed using Kleinberg’s algorithm, with the minimum duration of a burst set to 2 years.

## 3 Results

**Figure 1:**
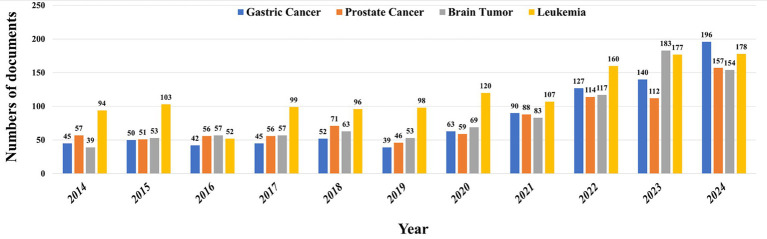
Trends in ferroptosis-related cancer research publications (2014–2024).

Figure 1 provides a bibliometric analysis of the annual publication output on research related to ferroptosis in gastric cancer, prostate cancer, brain tumors, and leukemia from 2014 to 2024, revealing the field’s clear evolutionary path from early exploration to recent explosive growth. During the initial phase from 2014 to 2020, the overall research output remained at a low and fluctuating level, with research interest in leukemia leading in most years, suggesting it may have been the first cancer type where ferroptosis research gained attention or achieved breakthroughs.

Beginning in 2021, the field reached a significant turning point, as the volume of literature on all four cancers started to grow rapidly, signaling that ferroptosis has gained widespread academic consensus as an important therapeutic target in oncology. Particularly noteworthy is the dynamic succession of research focus: during this growth period, interest in solid tumors comprehensively surpassed that in leukemia. Literature on brain tumors peaked in 2023 to take the lead, a trend potentially linked to the urgent clinical need to overcome their high treatment resistance. Subsequently, research on gastric cancer became the new frontrunner in 2024, reflecting its sustained research value as a cancer with high global incidence. Research on prostate cancer has also shown a strong and steady growth trend. This clear trajectory—from an early focus on hematological malignancies to a shift towards research on solid tumors driven by clinical challenges (especially brain and gastric cancers)—profoundly reflects that the field of ferroptosis is rapidly advancing from the elucidation of basic mechanisms to a new stage of clinically-oriented translational medicine research.

**Figure 2:**
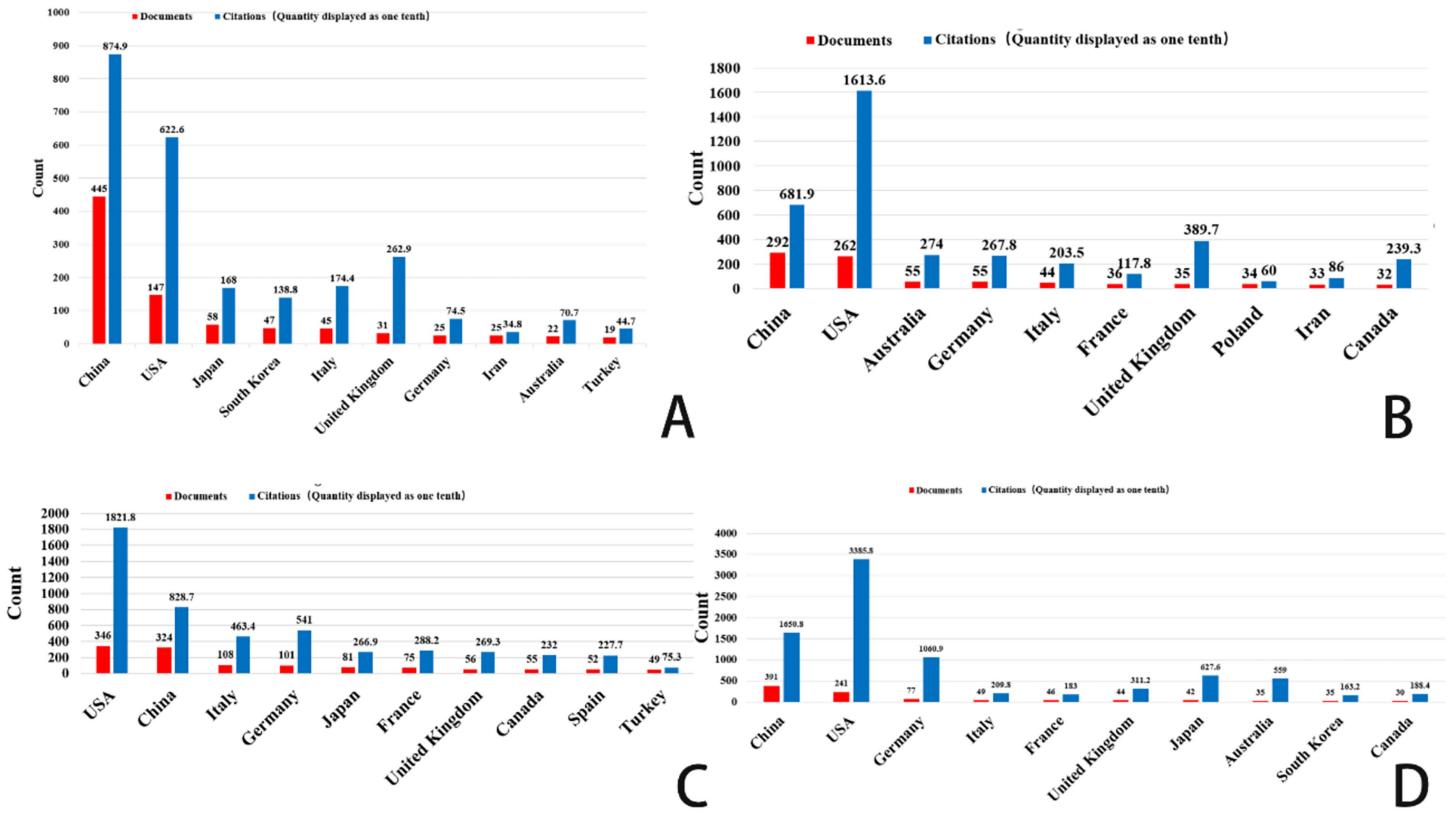
Top 10 countries/regions related to gastric cancer, prostate cancer, leukemia, and brain tumor. **(A)** Ferroptosis research in gastric cancer. **(B)** Ferroptosis research in prostate cancer. **(C)** Ferroptosis research in leukemia. **(D)** Ferroptosis research in brain tumors.

**Figure 3:**
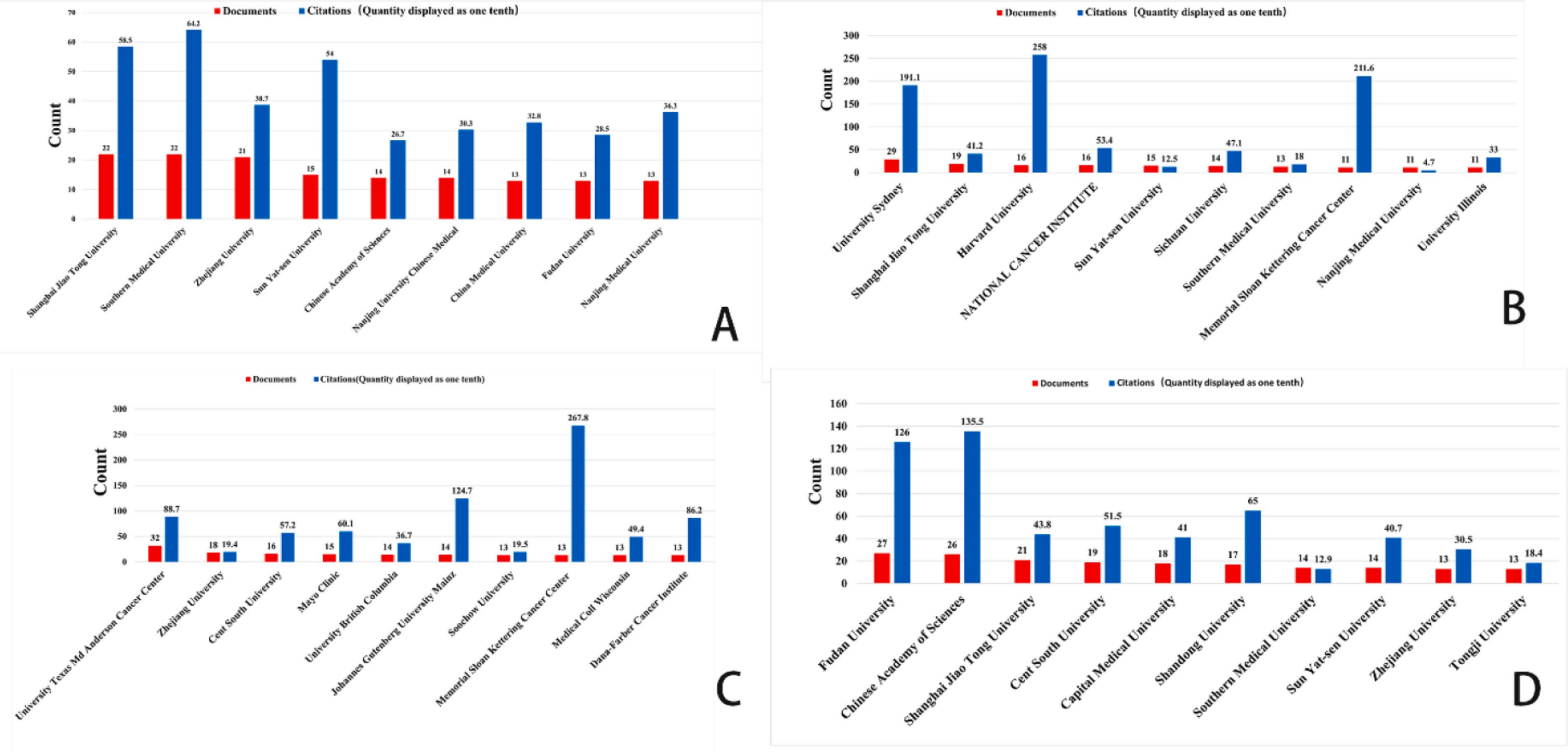
Top 10 organizations related to gastric cancer, prostate cancer, leukemia, and brain tumor. **(A)** Ferroptosis research in gastric cancer. **(B)** Ferroptosis research in prostate cancer. **(C)** Ferroptosis research in leukemia. **(D)** Ferroptosis research in brain tumors.

Figures 2, 3 shows that China had the highest publication output in gastric cancer, prostate cancer, and brain tumors during the study period. Specifically, China contributed 445 publications on gastric cancer and 391 on brain tumors. The average publication year for these documents from China is recent, suggesting high current research activity. Highly productive institutions from China, such as Shanghai Jiao Tong University, Southern Medical University, and Fudan University, feature prominently in the top 10 lists. Concurrently, institutions from the United States, including Vanderbilt University, the University of Sydney, and MD Anderson Cancer Center, also demonstrated significant research output. The data indicate that a large volume of research in these fields is concentrated in universities and academic institutions within China and the United States.

In this diagram, each circle represents an individual author. The size of the circle reflects the frequency with which the author’s work has been cited in the bibliographic coupling analysis. Larger circles indicate authors whose publications have been cited more frequently. The lines connecting the circles represent collaborative relationships between authors. The different colors within the network correspond to distinct research groups, highlighting the collaborative clusters formed by the authors.

**Figure 4:**
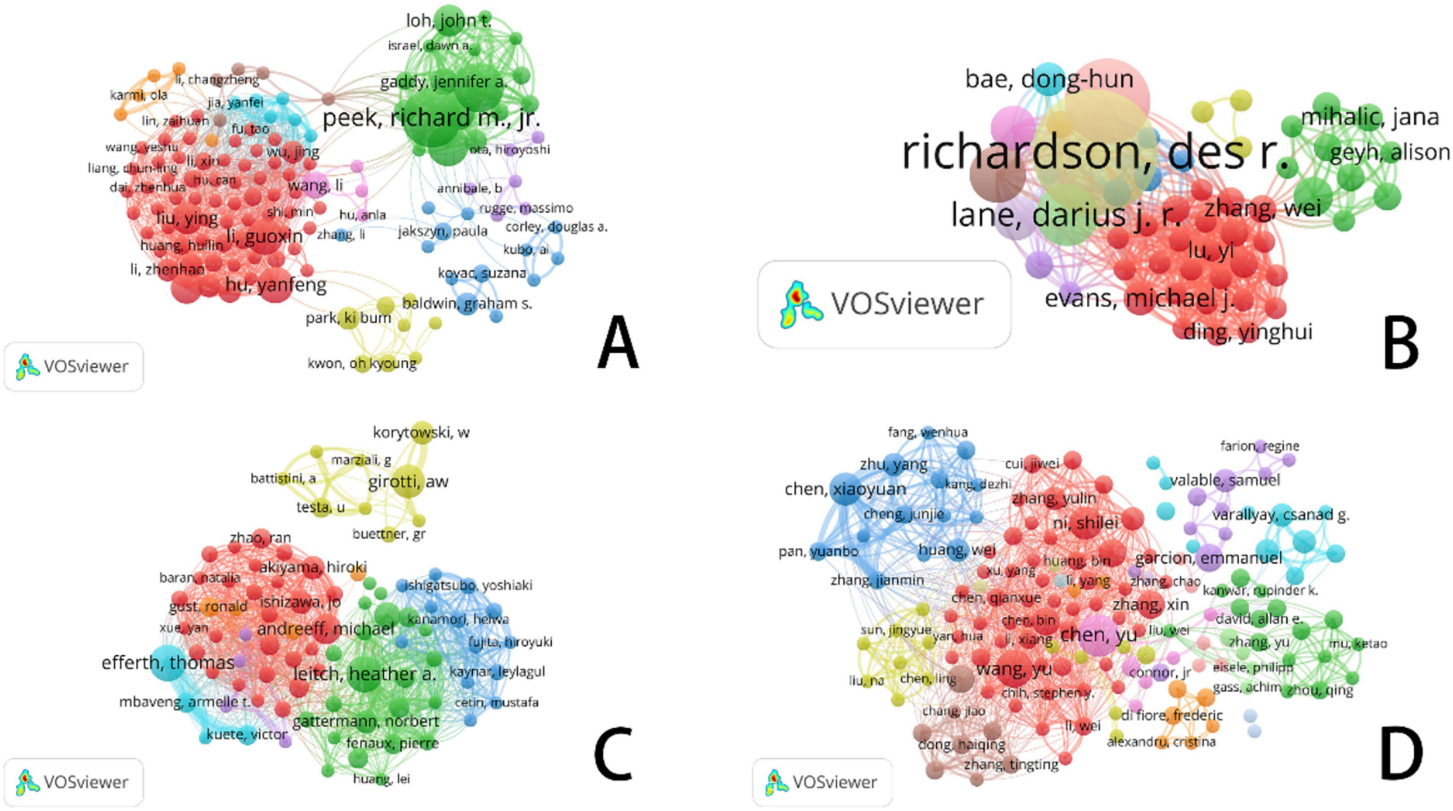
VOSviewer: collaborative networks of ferroptosis research authors in different cancer types (2014–2024). **(A)** Ferroptosis research in gastric cancer. **(B)** Ferroptosis research in prostate cancer. **(C)** Ferroptosis research in leukemia. **(D)** Ferroptosis research in brain tumors.

Figure 4 illustrates the authors’ analysis of studies related to ferroptosis in various cancer areas, providing insights into current research trends, collaboration networks, and potential future directions. The node sizes, color variations, and the connections between them help identify major research groups, key scholars, and emerging focal points within the research community.

In the area of gastric cancer, extensive research on ferroptosis has led to the formation of a complex network of authors. Figure 4A highlights several significant research groups, particularly a prominent green network led by Richard M. Jr., which underscores his influential leadership in this field. Other large collaborative groups further emphasize the role of ferroptosis as a regulator of tumor development and progression in gastric cancer, as well as its potential as a biomarker for diagnosis and treatment. The connections between different color groups, especially the red and green clusters, illustrate the practical implications of ferroptosis-related research in gastric cancer treatment, highlighting the importance of interdisciplinary collaboration in advancing clinical practices.

In contrast to gastric cancer, the volume of research on prostate cancer is smaller, but the core authors in this field—such as Richardson, Des R., Lane, Darius J. R., and Evans, Michael J.—show a more tightly-knit collaboration network (see Figure 4B). These scholars wield significant academic influence in the study of prostate cancer and ferroptosis. Unlike gastric cancer, the collaboration network among core authors in prostate cancer research is more compact, with fewer intersections between different author groups, resulting in a more concentrated research field. While cooperation among researchers is closer, interdisciplinary collaboration is relatively less prevalent.

In leukemia research (Figure 4C), the nodes within the research group are densely connected, indicating frequent academic cooperation among group members. This dense network highlights the strong influence of these research groups within the academic community. Compared to gastric and prostate cancer, leukemia research on ferroptosis has demonstrated relatively intensive academic exchanges and collaboration, though it remains in a developmental stage. The author cooperation network suggests that as recognition of the role of ferroptosis in leukemia treatment continues to grow, this field is likely to attract more emerging scholars, driving further research and the development of novel treatment approaches. With continued progress in clinical translational research and drug development, the application of ferroptosis in leukemia is expected to become a focal point in the near future.

In contrast to the other three cancer types, core authors in brain tumor research (Figure 4D), such as Chen Xiaoyuan, Chen Yu, and Wang Yu, exhibit a more pronounced collaborative relationship. Notably, within the red group, collaboration is particularly concentrated, reflecting a high level of academic synergy. When comparing the author coupling networks across the four cancer types, brain tumor research stands out for its high degree of interdisciplinary cooperation and internationalization, particularly in the study and application of ferroptosis mechanisms, which showcases strong innovation.

In the diagram, each circle represents an author’s citation frequency, with the size of the circle proportional to the number of times the author’s works have been referenced by others. Larger circles indicate a higher number of citations. The lines connecting the circles represent the relationships or collaborations between authors. Additionally, the color-coded networks signify distinct groups of authors who are likely collaborating or sharing similar research interests.

**Figure 5:**
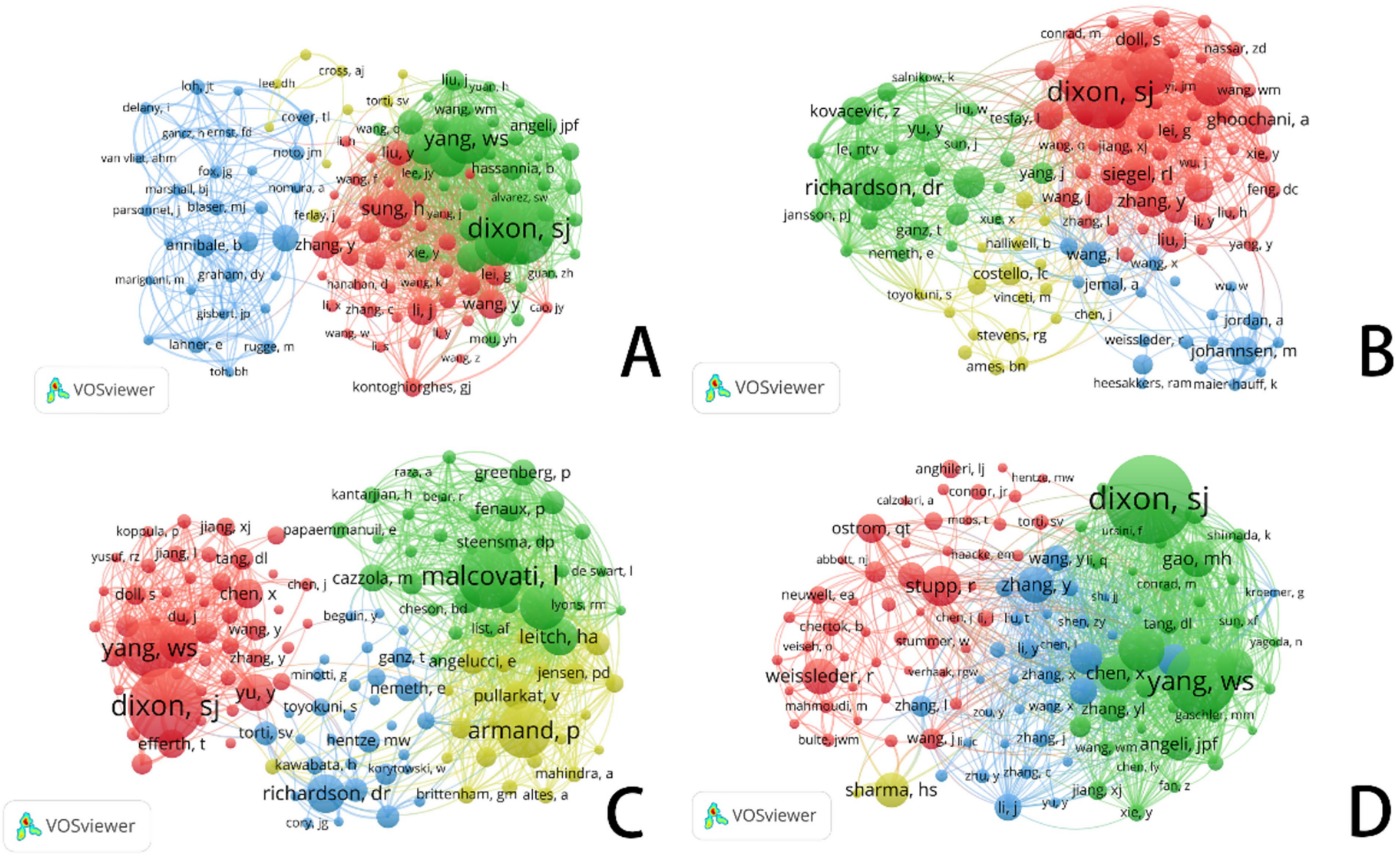
VOSviewer: co-citation network analysis of ferroptosis research authors in different cancer types. **(A)** Ferroptosis research in gastric cancer. **(B)** Ferroptosis research in prostate cancer. **(C)** Ferroptosis research in leukemia. **(D)** Ferroptosis research in brain tumors.

Figure 5 and Table 1 systematically categorize the relationships among scholars conducting ferroptosis-related research across four cancer types (gastric cancer, prostate cancer, leukemia, and brain tumors) through co-citation analysis. The co-citation network not only highlights researchers with significant academic influence in the field but also reflects the evolution of research hotspots, the emergence of new trends, and the establishment of academic collaborations.

**Table 1:** Top 10 authors and co-cited authors related to gastric cancer, prostate cancer, leukemia, and brain tumor.

Type	Rank	Author	Avg. pub. year	Documents	Citations	Rank	Co-cited author	Citations
Gastric cancer	1	Peek, Richard M. Jr.	2015	13	640	1	Dixon, S. J.	245
	2	Cover, Timothy L.	2015	11	522	2	Yang, W. S.	161
	3	Noto, Jennifer M.	2016	9	389	3	Chen, X.	133
	4	Hu, Yanfeng	2021	8	108	4	Sung, H.	122
	5	Li, Guoxin	2023	8	221	5	Stockwell, B. R.	115
	6	Chen, Zhian	2023	7	65	6	Zhang, H. Y.	105
	7	Liu, Ying	2022	7	215	7	Li, J.	90
	8	Loh, John T.	2016	7	261	8	Wang, Y.	89
	9	Piazuelo, M. Blanca	2016	7	377	9	Smyth, E. C.	88
	10	Gaddy, Jennifer A.	2015	6	350	10	Zhang, Y.	85
Prostate cancer	1	Richardson, Des R.	2014	22	1,386	1	Dixon, S. J.	176
	2	Kovacevic, Zaklina	2014	15	1,070	2	Yang, W. S.	113
	3	Lane, Darius J. R.	2016	11	794	3	Richardson, D. R.	102
	4	Jansson, Patric J.	2016	10	784	4	Stockwell, B. R.	91
	5	Kalinowski, Danuta S.	2015	8	630	5	Siegel, R. L.	77
	6	Sahni, Sumit	2015	8	652	6	Zhang, Y.	71
	7	Ivkov, Robert	2014	7	373	7	Torti, S. V.	70
	8	Bae, Dong-Hun	2017	6	311	8	Li, J.	65
	9	Evans, Michael J.	2019	5	120	9	Ghoochani, A.	64
	10	Antosiewicz, Jedrze J.	2009	5	304	10	Chen, X.	63
Leukemia	1	Effert, Thomas	2018	11	928	1	Dixon, S. J.	252
	2	Leitch, Heather A.	2017	11	228	2	Malcovati, L.	218
	3	Girotti, A. W.	1999	10	299	3	Yang, W. S.	189
	4	Andreeff, Michael	2020	9	85	4	Armand, P.	179
	5	Steensma, David P.	2012	8	211	5	Gattermann, N.	150
	6	Akiyama, Hiroki	2022	7	27	6	Richardson, D. R.	126
	7	Gattermann, Norbert	2014	7	368	7	Stockwell, B. R.	125
	8	Lshizawa, Jo	2022	7	27	8	Leitch, H. A.	120
	9	Korytowski, W.	2000	7	231	9	Yu, Y.	119
	10	Fenaux, Pierre	2014	6	448	10	Chen, X.	95
Brain tumor	1	Chen, Yu	2023	9	116	1	Dixon, S. J.	248
	2	Wang, Yu	2022	8	127	2	Yang, W. S.	196
	3	Chen, Xiaoyuan	2022	7	584	3	Stockwell, B. R.	128
	4	Ni, Shilei	2021	7	484	4	Stupp, R.	112
	5	Garcion, Emmanuel	2020	6	134	5	Doll, S.	103
	6	Li, Xingang	2020	6	432	6	Zhang, Y.	102
	7	Li, Yan	2020	6	636	7	Weissleder, R.	101
	8	Zhang, Wei	2020	6	157	8	Gao, M. H.	100
	9	Zhang, Xin	2019	6	189	9	Chen, X.	98
	10	Anghileri, L. J.	1999	5	38	10	Sharma, H. S.	97

In cancer research, academic activity related to ferroptosis is highly active, and its collaboration network exhibits a distinct centralized structure. Co-citation analysis reveals that Richard M. Jr. and Timothy L. Cover have made substantial contributions to this field. A significant portion of their work, primarily focused around 2015, has been cited over 500 times, emphasizing the far-reaching impact of their research. The blue cluster, centered around Annibale B. and Graham D. Y., underscores research on the tumor microenvironment, pathological mechanisms, and treatment strategies for gastric cancer.

In contrast, the red cluster, featuring researchers such as Dixon S. J., Yang W. S., and Sung H., primarily focuses on the mechanisms of cell death, particularly the role of ferroptosis in gastric cancer and other malignancies. Researchers in the green cluster are looking at ferroptosis’s therapeutic possibilities in stomach cancer, indicating a move towards precision medicine and the investigation of molecular processes.

Research on prostate cancer is increasingly stressing iron metabolism and ferroptosis pathways, which has clear relevance to gastric cancer. This change draws attention to notable developments in the identification of therapeutic targets and the development of precision medicine approaches. While research on prostate cancer is gradually encompassing the investigation of new cell death pathways, like ferroptosis, gastric cancer studies continue to emphasize pathogenic causes, early detection, and traditional treatments.

Influential academics like Dixon S. J., Siegel R. L., and Zhang Y. take centre stage inside the red cluster of prostate cancer research. Among them, Dixon S. J. distinguishes himself with his basic contributions to ferroptosis research, hence becoming a key player in changing the discipline. Focusing on iron metabolism, cell death processes, and the interaction between ferroptosis and the tumor microenvironment, the green cluster comprises academics such Richardson D. R. and Yu Y. Especially, Richardson D. R. has tightly connected iron metabolism to the survival and death of tumor cells, hence providing fresh insights for prostate cancer therapy.

Focusing on early findings in cancer metabolism and the molecular basis of ferroptosis, the yellow cluster includes scientists such Costello C. and Ames B. N. Bridging cell biology and biochemistry, their study highlights the importance of metabolic reprogramming and iron metabolism in cancer growth.

In treatment plans, researchers like Efferth Thomas and Leitch Heather A. have greatly helped to clarify the function of iron metabolism and ferroptosis. In recent years, increasing attention has been directed towards the mechanisms of drug resistance and relapse associated with ferroptosis in leukemia. As shown in Table 1, Dixon S. J. and Yang W. S. remain at the forefront of ferroptosis research, with their findings offering potential new targets for personalized leukemia therapy. The green cluster in Figure 5D is primarily composed of ferroptosis-focused researchers, with Dixon and Yang occupying central positions.

In brain tumor research, the blue and red clusters in Figure 5D illustrate growing interdisciplinary collaboration between ferroptosis and neuro-oncology. As tumor biology and cell death mechanisms converge, researchers such as Hang Y. and Gao M. H. are increasingly incorporating ferroptosis into studies of brain tumor molecular mechanisms. This integration reflects strong innovative potential in the field, signaling new directions for the understanding and treatment of brain tumors.

In this figure, each circle represents an academic journal. The size of each circle corresponds to the number of publications contributed by that journal in the bibliographic coupling analysis, with larger circles indicating higher publication volumes. The lines connecting the circles represent the strength of bibliographic coupling between journals. Color use emphasizes different groupings, hence reflecting cooperative trends and thematic links among the publications.

**Figure 6:**
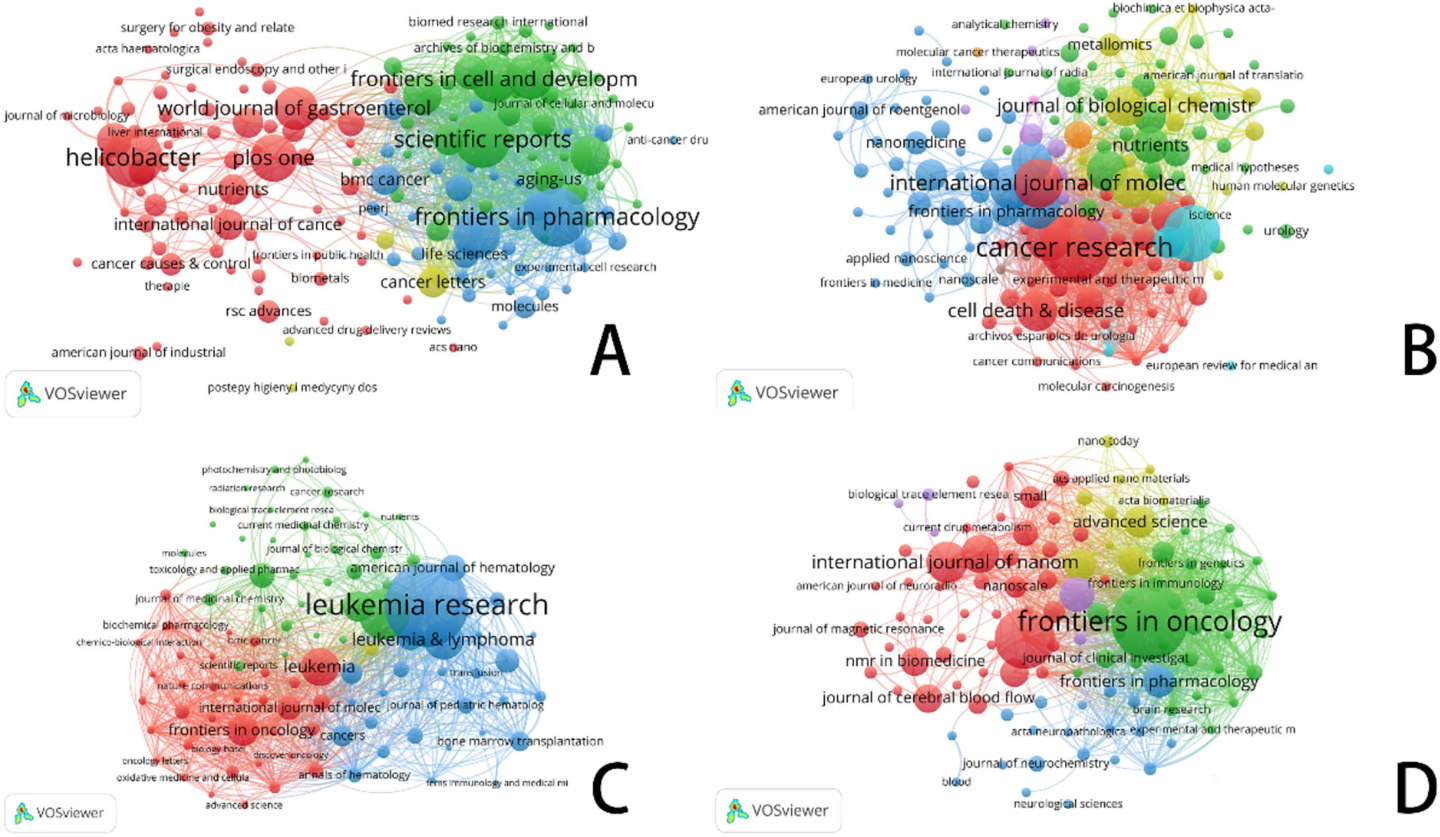
VOSviewer: visualization of bibliographic coupling in ferroptosis research across cancer types. **(A)** Ferroptosis research in gastric cancer. **(B)** Ferroptosis research in prostate cancer. **(C)** Ferroptosis research in leukemia. **(D)** Ferroptosis research in brain tumors.

**Figure 7:**
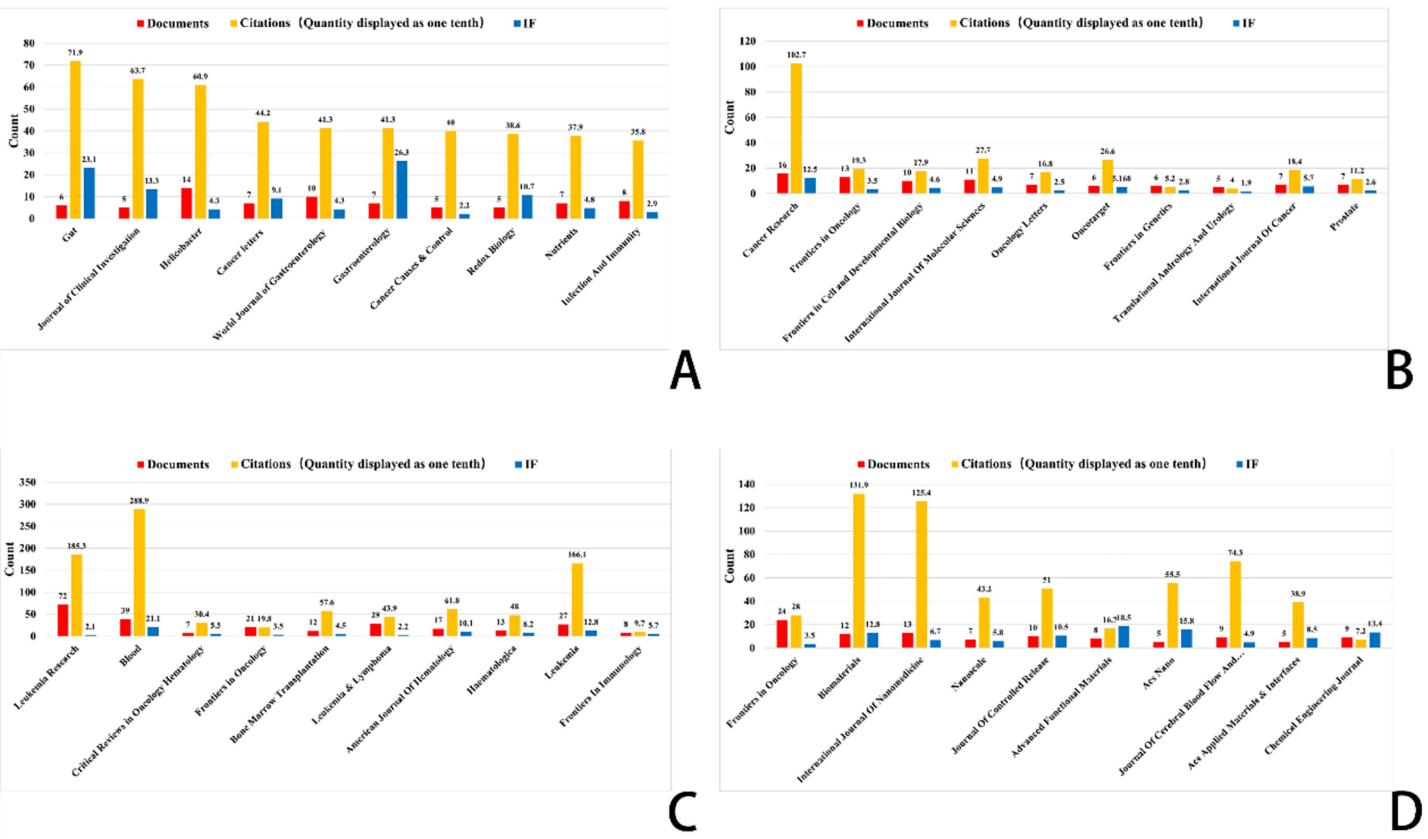
Top 10 journals related to gastric cancer, prostate cancer, leukemia, and brain tumor (Impact factor are sourced from the 2023 edition of the Journal Citation Reports (JCR) published by Clarivate). **(A)** Ferroptosis research in gastric cancer. **(B)** Ferroptosis research in prostate cancer. **(C)** Ferroptosis research in leukemia. **(D)** Ferroptosis research in brain tumors.

**Figure 8:**
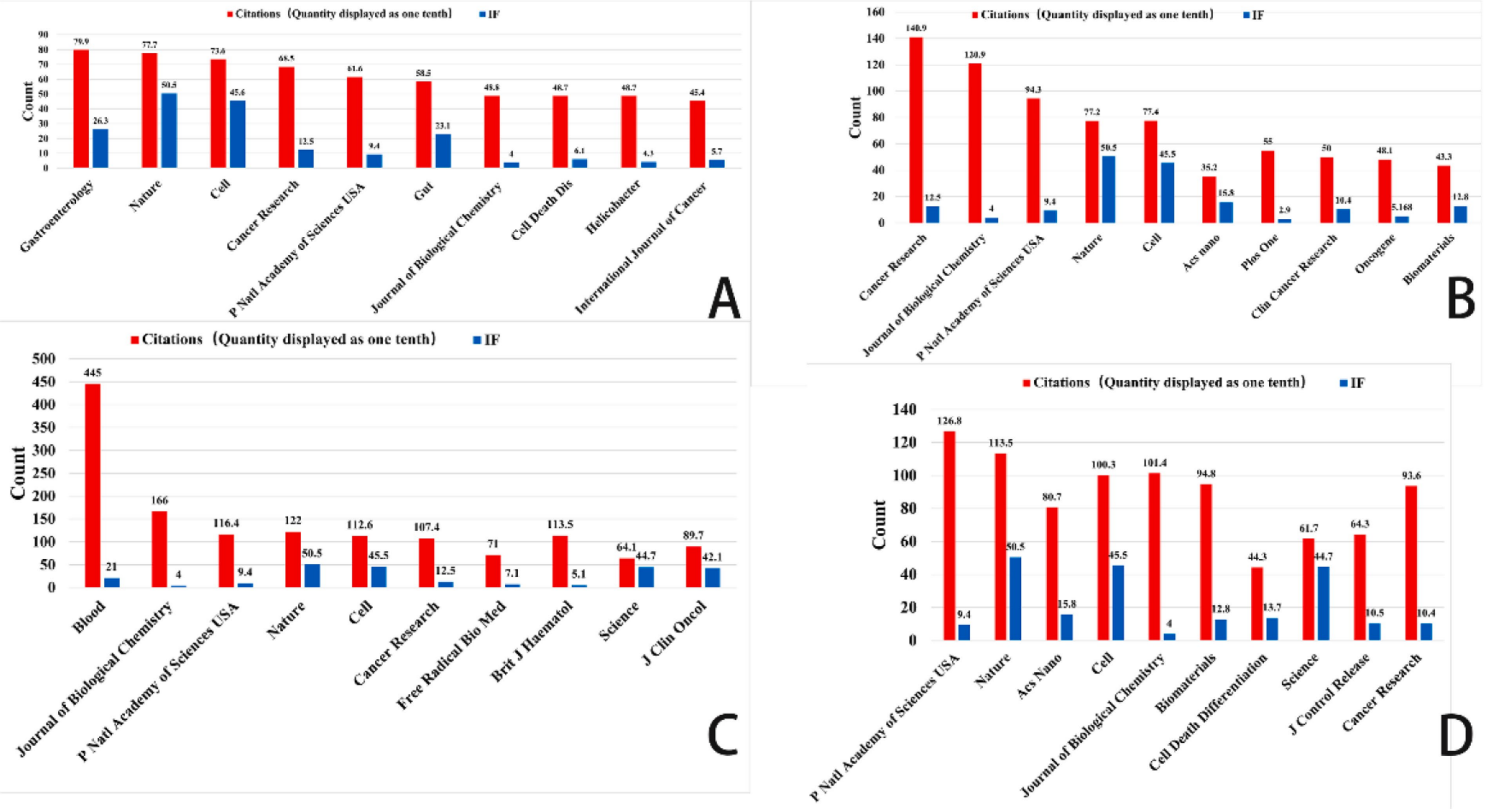
Top 10 co-cited journals related to gastric cancer, prostate cancer, leukemia, and brain tumor (Impact factor are sourced from the 2023 edition of the Journal Citation Reports (JCR) published by Clarivate). **(A)** Ferroptosis research in gastric cancer. **(B)** Ferroptosis research in prostate cancer. **(C)** Ferroptosis research in leukemia. **(D)** Ferroptosis research in brain tumors.

Focusing on important study fields connected to ferroptosis, Figures 6–8 offer a thorough analysis of how academic publications reference one another. These graphics show how several publications have helped to move ferroptosis research forward and underline the newest field trends. This paper emphasizes the need of ferroptosis in studies on gastric cancer, prostate cancer, leukemia, and brain tumors by means of bibliographic coupling analysis, hence showing the influence of prominent journals in these particular research areas.

The bibliographic coupling network in gastric cancer research shows an increasing trend of multidisciplinary cooperation. Among the most often referenced key journals in this area are Gut (impact factor 23.1), Journal of Clinical Investigation (impact factor 13.3), and Helicobacter (impact factor 4.3). In terms of citation impact, Gut emerged as the most influential journal with 719 citations, closely followed by the Journal of Clinical Investigation with 637 citations. This finding underscores the pivotal role these journals play as primary outlets for research on ferroptosis in gastric cancer.

This analysis identified the core journals in prostate cancer research to be Cancer Research (IF: 12.5), Frontiers in Oncology (IF: 3.5), and the International Journal of Molecular Sciences (IF: 4.9). Garnering 1,027 citations, Cancer Research not only emerges as the foremost journal in terms of citation impact but also underscores its prominent status within the field. Analysis of bibliographic coupling networks reveals that prostate cancer research includes significant multidisciplinary cooperation and the use of sophisticated technology. Future studies in this field will probably stress the creation of new methods in molecular targeting, precision medicine, and nanotechnology.

Bibliographic coupling analysis in leukemia research emphasizes the wide range of fields involved—hematology, oncology, molecular biology, immunology, and pharmacology among others. Leukemia’s relationship with lymphoma and bone marrow transformation closely parallels its overlap with other hematologic disorders. Key publications like Journal of Medicinal Chemistry and Frontiers in Oncology show the close relationships between treatment approaches, drug development, and leukemia research. Future studies will concentrate on molecular targets, drug development, immunotherapy, and individualized treatment strategies, all of which are anticipated to increase patient outcomes and treatment alternatives.

Key publications in brain tumor studies are Frontiers in Oncology, Biomaterials, and the International Journal of Nanomedicine. These publications highlight important research topics like molecular processes, immunotherapy, and nanotechnology. Research on brain tumors covers several fields including gene alterations, cancer processes, immune evasion, nano drug delivery, early diagnostic biomarkers, and stem cell therapies. The general trend shows that brain tumor research is moving towards multidisciplinary integration, precision tailored therapy, and creative immunotherapy. Future treatment approaches are expected to increasingly rely on advanced technologies, such as nanotechnology and gene editing, which are poised to drive breakthroughs in treatment and significantly enhance patient prognosis.

Each circle in the diagram represents an individual document. The size of the circle reflects the number of citations that the document has received, with larger circles indicating more frequently cited documents. The lines connecting the circles represent the relationships between documents, showing how they are linked. The color-coded networks identify collaboration clusters, which highlight groups of documents that are closely related and form cooperative networks in the scholarly literature.

**Figure 9:**
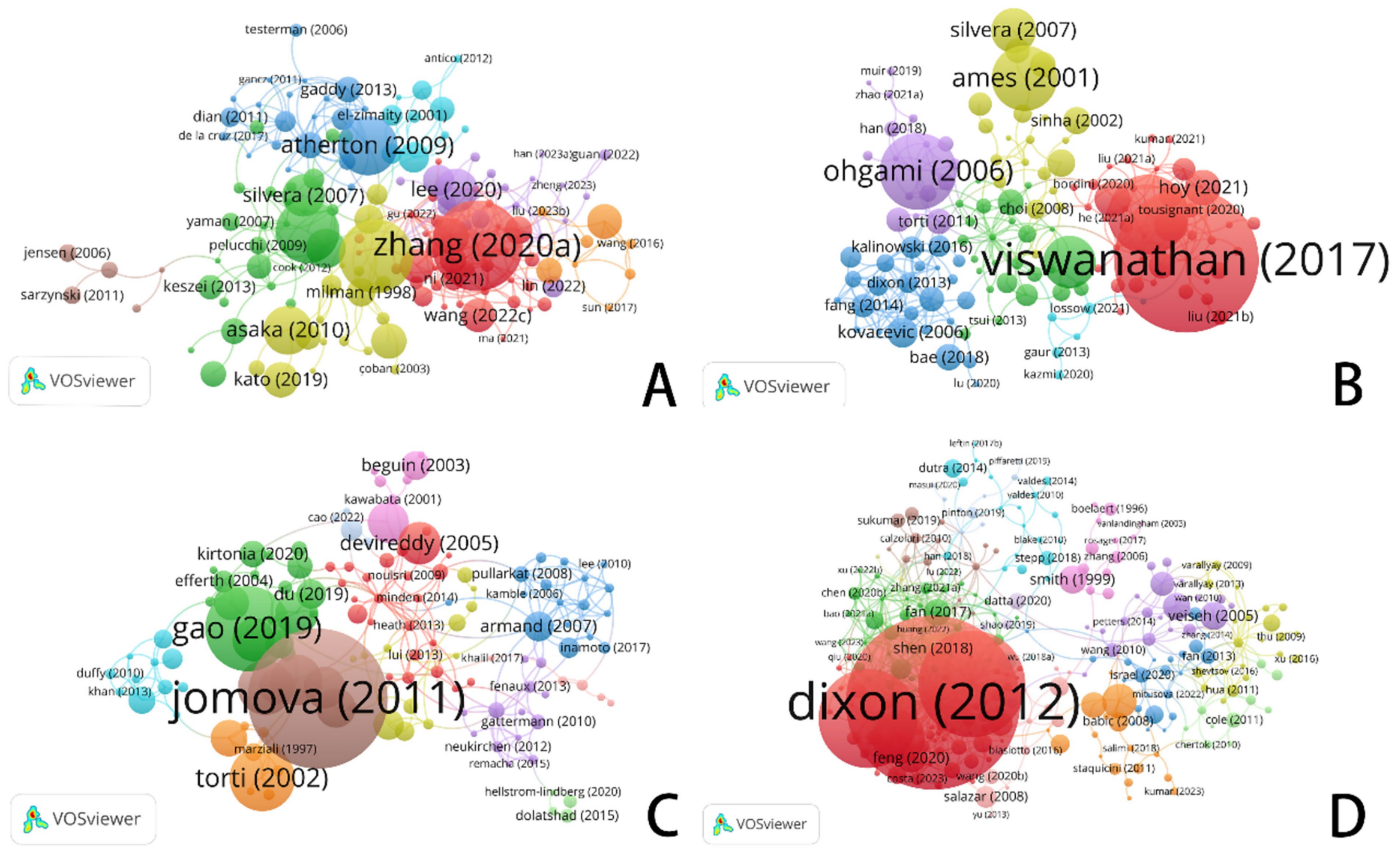
VOSviewer: visualization of bibliographic coupling networks in ferroptosis research across cancer types. **(A)** Ferroptosis in gastric cancer. **(B)** Ferroptosis in prostate cancer. **(C)** Ferroptosis in leukemia. **(D)** Ferroptosis in brain tumors.

An examination of frequently cited literature provides valuable insights into emerging research topics, trends, and potential clinical applications of ferroptosis in various types of cancer (refer to Figure 9). In the following section, I will explore how ferroptosis plays a role in cancers such as gastric cancer, prostate cancer, leukemia, and brain tumors, with a particular focus on the most recent advancements in these areas.

Regarding gastric cancer, bibliometric analysis highlights significant works by Zhang (2020a) and Atherton (2009). Zhang’s (2020a) study explored the involvement of ferroptosis in either supporting or inhibiting the survival of gastric cancer cells. In contrast, Gaddy (2013) and Atherton (2009) investigated the relationship between iron deficiency—often caused by infection with *Helicobacter pylori*—and its role in gastric cancer. This research underscores how ferroptosis influences the growth and transformation of gastric cancer cells through cellular metabolism.

The guidelines proposed by Asaka (2010) and Kato (2019) for managing *Helicobacter pylori* infection have made substantial contributions to the prevention and treatment of gastric cancer, as evidenced by their extensive citation in clinical practice. These foundational studies have established the fundamental groundwork for investigating the mechanisms underlying iron metabolism and cell death, thereby catalyzing subsequent advancements in ferroptosis research within the context of gastric cancer.

Among the most frequently cited and influential publications in prostate cancer research are the seminal works of Viswanathan (2017) and Yi (2020). By elucidating how ferroptosis alters prostate cancer cell activity, this study underscores the pivotal role of lipid peroxidation in mediating both treatment resistance and the ferroptotic process itself. The study by Yi (2020) revealed that modulating lipid synthesis via the PI3K-Akt-mTOR signaling pathway can suppress ferroptosis, presenting a novel therapeutic avenue for cancer treatment. Ohgami’s 2006 study provided foundational insights into the intricate link between iron metabolism and a distinct form of iron-dependent cell death, a process now recognized as ferroptosis. It dug into ladder proteins’ role. These steer iron metabolism. They sway ferroptosis in prostate cancer too. A deeper understanding of the intricate interplay among ferroptosis, lipid peroxidation, and iron metabolism provides a strong rationale for developing therapeutic strategies that target these pathways to overcome drug resistance and induce cell death in prostate cancer.

The recommendations put forth by Asaka (2010) and Kato (2019) for the management of *Helicobacter pylori* infection are foundational to modern strategies for the prevention and treatment of gastric cancer. The enduring relevance of these studies is underscored by their frequent citation within contemporary therapeutic guidelines and clinical research. These studies provide a comprehensive framework for understanding the mechanisms governing cell death and iron metabolism, thereby catalyzing further advancements in ferroptosis research within the context of gastric cancer.

The seminal works by Viswanathan (2017) and Yi (2020) have been pivotal, demonstrating the regulatory function of ferroptosis in prostate cancer cells and establishing lipid peroxidation as the central mechanism governing both treatment resistance and cell death. By linking the PI3K-Akt-mTOR pathway’s promotion of lipid synthesis to the suppression of ferroptosis, Yi (2020) provided a strong rationale for a new therapeutic strategy: inhibiting this pathway to dismantle cancer cells’ defenses and trigger cell death. Our findings demonstrate that prostate cancer cell viability and chemoresistance are directly modulated by ferroptosis activation, with iron metabolism and lipid peroxidation serving as key determinants. Specifically, dysregulation of iron homeostasis and *de novo* lipogenesis significantly influences therapeutic outcomes, suggesting actionable targets for precision oncology.

Dixon (2012) and Stockwell (2017) made groundbreaking contributions to elucidating the mechanisms of ferroptosis in brain tumors. Dixon’s seminal 2012 study laid the foundation for subsequent brain tumor research by elucidating the fundamental mechanisms of ferroptosis. Stockwell (2017) advanced the understanding of the role of ferroptosis in brain cancers by investigating its interplay with metabolic processes and reactive oxygen species (ROS), thereby revealing potential therapeutic strategies.

The studies by Jomova (2011) and Gao (2019) have provided novel insights into the mechanisms of iron-dependent cell death in leukemia. The mechanistic focus of these studies differs: Gao (2019) centered on the involvement of mitochondria, while Jomova (2011) underscored the induction of leukemic cell death through iron-driven oxidative stress. The findings from Gao (2019) suggest that modulating mitochondrial iron metabolism may represent a viable therapeutic strategy for leukemia. Modulating the homeostasis of intracellular iron ions to regulate iron-dependent cell death could offer a novel therapeutic approach for leukemia. Modifications in metabolic pathways that cause iron-dependent cell death in brain tumors might perhaps offer chances to inhibit tumor cell proliferation.

Every circle in this figure is a reference; the size of the circle corresponds to the number of co-citations linked to that reference. Larger rings show references that have been co-cited more often. The lines connecting the circles represent the relationships between references, while the color-coded clusters highlight collaborative groups, demonstrating the interconnectedness of references within each group.

**Figure 10:**
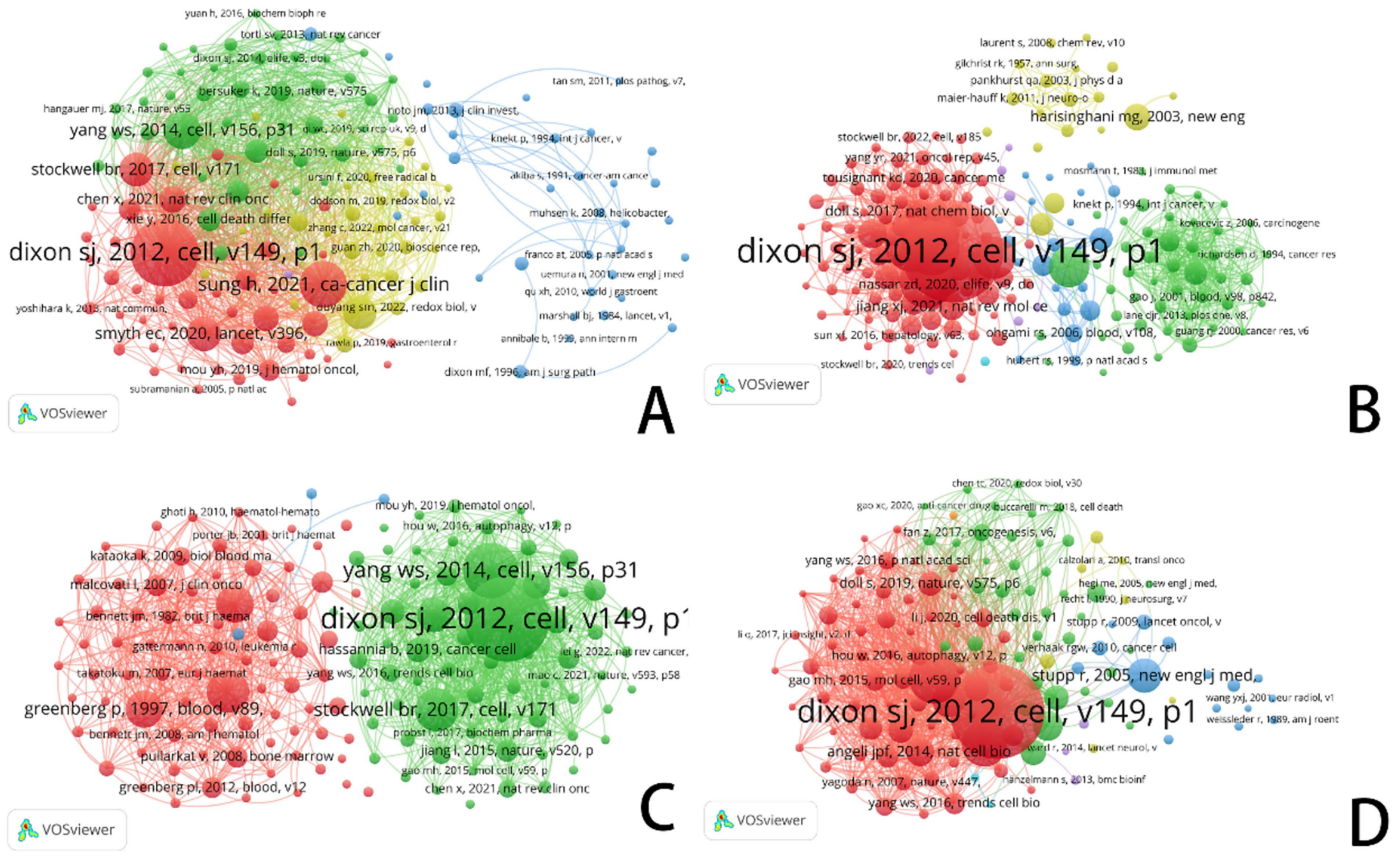
VOSviewer: visualization of co-citation reference networks in ferroptosis research across cancer types. **(A)** Ferroptosis research in gastric cancer. **(B)** Ferroptosis research in prostate cancer. **(C)** Ferroptosis research in leukemia. **(D)** Ferroptosis research in brain tumors.

Through co-citation analysis, Dixon (2012), Viswanathan (2017), and Jomova (2011) have emphasized the critical role of iron metabolism and iron-dependent cell death across various cancer types. The gathering of iron ions and the process of lipid peroxidation are usual mechanisms that lead to iron-dependent cell demise in many types of cancer. The study of different kinds of cancer mostly looks at how to control things like iron metabolism, the making of lipids, and how mitochondrias work. The goal is to either encourage or stop cell death that relies on iron which could bring about new ways to treat cancers. In the future, studies might dig deeper into finding exact ways to manage this iron-dependent cell death in diverse cancer types, opening doors for major advances in the treatment of patients (refer to Figure 10).

**Figure 11:**
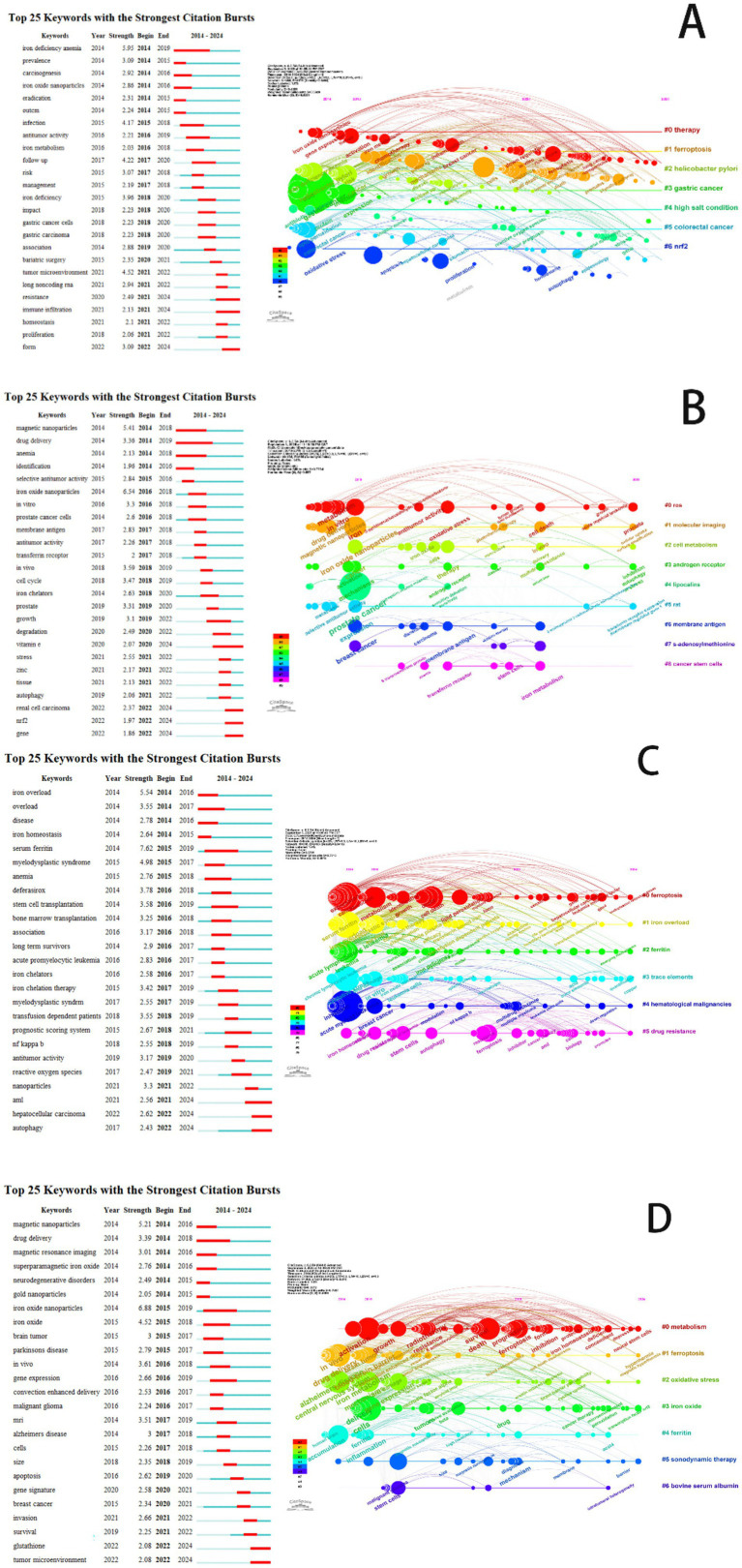
A keyword co-occurrence network visualizing research hotspots in the field of ferroptosis. **(A)** Ferroptosis research in gastric cancer. **(B)** Ferroptosis research in prostate cancer. **(C)** Ferroptosis research in leukemia. **(D)** Ferroptosis research in brain tumors.

To identify the evolving research frontiers and emerging hotspots in ferroptosis research for each cancer type, we conducted a citation burst analysis of keywords (Figure 11). This analysis pinpoints keywords that have received a surge of attention, with red bars indicating the duration of a burst and those extending to 2024 signifying current research frontiers. The findings reveal distinct, cancer-specific trajectories. For gastric cancer, the research is strongly linked to etiology, with powerful bursts for keywords “*Helicobacter pylori*,” “high salt condition,” and “iron deficiency anemia,” suggesting a focus on connecting these risk factors to ferroptosis induction and therapeutic resistance. In prostate cancer research, a dual focus emerged, highlighting the integration of ferroptosis with core signaling pathways through the “androgen receptor” alongside a significant trend towards translational nanomedicine, evidenced by bursts in “magnetic nanoparticles” and “drug delivery.” The landscape for leukemia is uniquely characterized by its clinical focus on systemic iron metabolism, where the most potent bursts are associated with “iron overload,” “serum ferritin,” and “iron chelation therapy,” reflecting efforts to understand ferroptosis in the context of treatment-related iron dysregulation. Research on brain tumors is unequivocally dominated by the challenge of therapeutic delivery; keywords with significant bursts, “blood–brain barrier,” “drug delivery,” and the emerging “sonodynamic therapy,” indicate that the primary frontier involves engineering innovative strategies to transport ferroptosis-inducing agents to the tumor site. The keyword burst analysis provides a dynamic view of these research frontiers, showing a clear progression from foundational concepts towards tailored clinical applications shaped by the unique etiology, signaling pathways, systemic pathophysiology, and anatomical barriers of each malignancy.

## 4 Discussion

This bibliometric analysis provides a comprehensive cartography of the rapidly evolving landscape of ferroptosis research across gastric cancer, prostate cancer, leukemia, and brain tumors from 2014 to 2024. Beyond merely quantifying publication output, our study leverages keyword burst detection and co-citation network analysis to illuminate the shifting thematic contours and intellectual turning points. The significant surge in publications, particularly the pronounced increase in gastric cancer research, signals a maturation of the field from foundational discovery towards translational application. The emergence and persistence of keywords such as “therapy resistance,” “tumor microenvironment,” “immunotherapy,” and “nanotechnology” across these diverse malignancies strongly indicate that the research community is strategically exploring ferroptosis as a means to address recalcitrant clinical challenges. This reflects a growing consensus to leverage ferroptosis strategically, transforming it from a biological curiosity into a tool designed to circumvent the shortcomings of conventional treatments.

### 4.1 The evolving mechanistic focus: from core biochemistry to regulatory complexity

Our co-citation analysis reveals that the field’s intellectual foundation is built upon seminal studies that established ferroptosis as a distinct form of regulated cell death, defined by its core mechanism of iron-dependent lipid peroxidation. The earliest keyword clusters reveal that the field’s nascent research efforts were predominantly focused on delineating the fundamental biology of this process. Our keyword burst detection reveals a sophisticated evolution. While foundational principles, such as oxidative stress and redox dysregulation, provide the underlying conceptual framework, investigative efforts are now increasingly directed towards the specific molecular machinery governing this pathway.

The enduring prominence of “GPX4” as a keyword reflects its established identity as the master regulator of ferroptosis, safeguarding cells from lethal lipid peroxidation by maintaining redox balance. The recent emergence of keywords “ACSL4,” “FSP1,” and “p53” indicates a significant deepening of the field’s inquiry, moving beyond core mechanisms to explore a more complex regulatory network. This progression reflects the maturation of the field, as the central inquiry shifts from characterizing the canonical pathway of ferroptosis to dissecting its complex, context-dependent regulatory networks. ACSL4 promotes the incorporation of polyunsaturated fatty acids (PUFAs) into cellular membranes, thereby sensitizing them to lipid peroxidation, while FSP1 mediates a parallel protective mechanism that functions independently of GPX4. This transition from a GPX4-centric view to a broader network perspective signifies the field’s maturing understanding of ferroptosis as a process governed by a multi-layered and redundant regulatory system, acknowledging a complexity that extends far beyond iron dysregulation alone.

### 4.2 The translational pivot: harnessing ferroptosis to overcome therapeutic bottlenecks

The marked increase in the prominence of “therapy resistance” as a keyword serves as a definitive indicator of the field’s translational trajectory, highlighting its ambition to tackle critical clinical challenges. This trend is indicative of a concerted effort to leverage the distinct mechanism of ferroptosis as a means to target cancer cell populations that have become refractory to apoptosis-inducing therapies. Herein lies the profound clinical potential of ferroptosis: it directly addresses therapeutic resistance by initiating a distinct, non-apoptotic cell death program. This program, driven by rampant lipid peroxidation, effectively circumvents the resistance pathways that neutralize conventional chemotherapies and targeted agents.

The simultaneous emergence of “immunotherapy” signifies a paradigm shift towards combination strategies. Mechanistically, the accumulation of lipid peroxides during ferroptosis can lead to the release of immunogenic signals (DAMPs), thereby converting an immunologically “cold” tumor into a “hot” one that is more responsive to immune checkpoint blockade. Our bibliometric data provides a clear footprint of this conceptual convergence. The future clinical scope, therefore, is not centered on ferroptosis as a monotherapy, but as a synergistic partner to enhance the efficacy of existing treatments. The parallel rise of “nanotechnology” directly supports this vision by offering a tangible solution to deliver ferroptosis inducers specifically to tumor sites, minimizing systemic toxicity and maximizing therapeutic impact.

### 4.3 Future directions: cancer-specific vulnerabilities and organ-specific challenges

While unified by core mechanisms, the research trajectories across the four cancers reveal distinct priorities that shape the future research landscape. For solid tumors like gastric and prostate cancer, the “tumor microenvironment” (TME) remains a key frontier. Future research must dissect how the unique metabolic state of the TME alters the cellular redox balance and lipid metabolism, thereby creating specific ferroptotic vulnerabilities to be exploited.

For brain tumors, the primary challenge remains the blood–brain barrier (BBB). The keyword trends underscore that future breakthroughs will depend not only on novel ferroptosis inducers but also on innovative delivery platforms that can achieve therapeutic concentrations in the brain. For leukemia, where the concept of a solid TME is less relevant, future studies will likely focus on intrinsic metabolic dependencies and iron handling pathways unique to hematopoietic malignancies.

Our analysis highlights a clear path forward, indicating that the next wave of research will likely evolve along three interconnected fronts. A primary focus will be on precision targeting, aiming to identify biomarkers that can predict patient response to ferroptosis-based therapies. A deeper understanding of mechanistic interplay will guide the design of rational synergistic combinations, with a particular emphasis on immunotherapy. Conquering biological obstacles such as the BBB will necessitate the development of advanced delivery technologies. This version uses more formal language and a slightly more detailed structure, making it a robust and authoritative concluding statement.

## 5 Advantages and limitations of research

The primary strength of this study is derived from the novelty of its research topic combined with the comprehensive and systematic nature of its analysis. This work systematically analyzes the scholarly trajectory and key developments in ferroptosis research across four clinically significant malignancies (gastric cancer, prostate cancer, leukemia, and brain tumors) over the period of 2014–2024. Leveraging a comprehensive dataset from the Web of Science Core Collection, Scopus, and PubMed, and employing established bibliometric tools (VOSviewer, CiteSpace), this study systematically maps the intellectual landscape of the field. The analysis quantifies publication trends, identifies the most productive countries, institutions, and authors, delineates collaboration networks, and pinpoints the most influential journals. The keyword emergence analysis pinpointed nascent research directions, notably the integration of immunotherapy with ferroptosis inducers and the development of nanotechnology-based therapeutic strategies. At the same time, it distinguished the research focus of different cancer species, and tried to associate bibliometric indicators with potential biological mechanism exploration and clinical application prospects, providing a valuable multi-dimensional reference framework and empirical basis for future research in this field.

The limitations of this study also need to be clearly pointed out. The data source is single, which may lead to selective bias, and the relevant high-quality studies not included in the database or published in non-English are omitted. Bibliometrics essentially focuses on quantitative indicators (such as the number of publications and citations), and it is difficult to conduct in-depth qualitative evaluation of the internal scientific quality of literature, the rigor of research design or the reliability of conclusions. The research results may be affected by the “citation delay” effect, that is, the influence of newly published important research may be underestimated because it has not accumulated enough citations, and the comprehensiveness and accuracy of the analysis are limited by the coverage of the selected key words, which may not fully capture all relevant concepts or emerging terms. Although this study explores the mechanism and clinical application prospects, these are mainly based on the inference of literature publishing mode, rather than direct experimental verification or systematic evaluation of clinical trial data. The extrapolation of research conclusions is limited by the four specific cancer types selected.

## 6 Conclusion

This study conducts a systematic analysis of the mechanisms of ferroptosis in gastric cancer, prostate cancer, leukemia, and brain tumors using bibliometric methods. It highlights the therapeutic potential and emerging trends of ferroptosis in these cancers. The early paradigm for ferroptosis mechanisms was established in gastric cancer research, paving the way for expanding studies on prostate cancer, leukemia, and brain tumors. This suggests the vast potential of ferroptosis as an innovative therapeutic target.

Despite considerable progress in both basic and clinical research on the underlying mechanisms of ferroptosis, several challenges persist in translating these findings into clinical applications. To drive further advancements in the field, it is crucial to strengthen interdisciplinary collaboration and clinical validation. These efforts will be key to advancing drug development targeting the ferroptosis mechanism, enabling early detection of dysregulated iron metabolism, and fostering the development of personalized treatment strategies. This study provides a scientific foundation for the application of the ferroptosis mechanism in cancer therapy and offers guidance for subsequent clinical research and pharmaceutical development. As related technologies and research progress, ferroptosis is poised to emerge as an innovative strategy in cancer treatment, potentially offering patients a broader range of therapeutic options and improving prognostic outcomes.

## Data Availability

The original contributions presented in the study are included in the article/Supplementary material, further inquiries can be directed to the corresponding authors.

## References

[1] Xu X Zhou N Lan H Yang F Dong B Zhang X. The ferroptosis molecular subtype reveals characteristics of the tumor microenvironment, immunotherapeutic response, and prognosis in gastric cancer. Int J Mol Sci. (2022) 23:9767. doi: 10.3390/ijms23179767, PMID: 36077165 PMC9456108

[2] Shahid W Iqbal A Iqbal I Mehmood A Jia H. Application of ferroptosis strategy to overcome tumor therapy resistance in breast and different cancer cells. Iran J Basic Med Sci. (2024) 27:1085–95. doi: 10.22038/IJBMS.2024.77465.16752, PMID: 39055871 PMC11266745

[3] Yu Y Yan Y Niu F Wang Y Chen X Su G . Ferroptosis: a cell death connecting oxidative stress, inflammation and cardiovascular diseases. Cell Death Discov. (2021) 7:193. doi: 10.1038/s41420-021-00579-w, PMID: 34312370 PMC8313570

[4] Jenke R Oliinyk D Zenz T Körfer J Schäker-Hübner L Hansen FK . HDAC inhibitors activate lipid peroxidation and ferroptosis in gastric cancer. Biochem Pharmacol. (2024) 225:116257. doi: 10.1016/j.bcp.2024.116257, PMID: 38705532

[5] Xi J Tian LL Xi J Girimpuhwe D Huang C Ma R . Alterperylenol as a novel thioredoxin reductase inhibitor induces liver cancer cell apoptosis and ferroptosis. J Agric Food Chem. (2022) 70:15763–75. doi: 10.1021/acs.jafc.2c05339, PMID: 36472370

[6] Kajarabille N Latunde-Dada GO. Programmed cell-death by ferroptosis: antioxidants as mitigators. Int J Mol Sci. (2019) 20:4968. doi: 10.3390/ijms20194968, PMID: 31597407 PMC6801403

[7] Bertrand RL. Iron accumulation, glutathione depletion, and lipid peroxidation must occur simultaneously during ferroptosis and are mutually amplifying events. Med Hypotheses. (2017) 101:69–74. doi: 10.1016/j.mehy.2017.02.017, PMID: 28351498

[8] Ouyang S Li H Lou L Huang Q Zhang Z Mo J . Inhibition of STAT3-ferroptosis negative regulatory axis suppresses tumor growth and alleviates chemoresistance in gastric cancer. Redox Biol. (2022) 52:102317. doi: 10.1016/j.redox.2022.102317, PMID: 35483272 PMC9108091

[9] Wu X Chen S Huang K Lin G. Triptolide promotes ferroptosis by suppressing Nrf2 to overcome leukemia cell resistance to doxorubicin. Mol Med Rep. (2023) 27:17. doi: 10.3892/mmr.2022.12904, PMID: 36453238 PMC9743397

[10] Jiang X Huang Y Hong X Wu W Lin Y Lin L . Exogenous dihomo-γ-linolenic acid triggers ferroptosis via ACSL4-mediated lipid metabolic reprogramming in acute myeloid leukemia cells. Transl Oncol. (2025) 52:102227. doi: 10.1016/j.tranon.2024.102227, PMID: 39644823 PMC11667188

[11] Ashoub MH Razavi R Heydaryan K Salavati-Niasari M Amiri M. Targeting ferroptosis for leukemia therapy: exploring novel strategies from its mechanisms and role in leukemia based on nanotechnology. Eur J Med Res. (2024) 29:224. doi: 10.1186/s40001-024-01822-7, PMID: 38594732 PMC11003188

[12] Zheng F Wang Y Zhang Q Chen Q Liang CL Liu H . Corrigendum: Polyphyllin I suppresses the gastric cancer growth by promoting cancer cell ferroptosis. Front Pharmacol. (2023) 14:1201715. doi: 10.3389/fphar.2023.1201715, PMID: 37205909 PMC10191630

[13] Jiang M Qiao M Zhao C Deng J Li X Zhou C. Targeting ferroptosis for cancer therapy: exploring novel strategies from its mechanisms and role in cancers. Transl Lung Cancer Res. (2020) 9:1569–84. doi: 10.21037/tlcr-20-341, PMID: 32953528 PMC7481593

[14] Cheng L He Q Liu B Chen L Lv F Li X . SGK2 promotes prostate cancer metastasis by inhibiting ferroptosis via upregulating GPX4. Cell Death Dis. (2023) 14:74. doi: 10.1038/s41419-023-05614-5, PMID: 36720852 PMC9889330

[15] Wei G Huang Y Li W Xie Y Zhang D Niu Y . SREBF1-based metabolic reprogramming in prostate cancer promotes tumor ferroptosis resistance. Cell Death Discov. (2025) 11:75. doi: 10.1038/s41420-025-02354-7, PMID: 39988626 PMC11847930

[16] Chen H Lyu F Gao X. Advances in ferroptosis for castration-resistant prostate cancer treatment: novel drug targets and combination therapy strategies. Prostate Cancer Prostatic Dis. (2024). doi: 10.1038/s41391-024-00933-w, PMID: 39733054 PMC12909126

[17] Chen X Pang X Yeo AJ Xie S Xiang M Shi B . The molecular mechanisms of ferroptosis and its role in blood-brain barrier dysfunction. Front Cell Neurosci. (2022) 16:889765. doi: 10.3389/fncel.2022.889765, PMID: 35663422 PMC9160190

[18] Xiong J Qi W Liu J Zhang Z Wang Z Bao J . Research progress of ferroptosis: a bibliometrics and visual analysis study. J Healthc Eng. (2021) 2021:2178281. doi: 10.1155/2021/2178281 34413966 PMC8370827

[19] Cheng K Guo Q Shen Z Yang W Zhou Y Sun Z . Frontiers of ferroptosis research: an analysis from the top 100 most influential articles in the field. Front Oncol. (2022) 12:948389. doi: 10.3389/fonc.2022.948389, PMID: 36033530 PMC9403769

[20] Maremonti F Tonnus W Gavali S Bornstein S Shah A Giacca M . Ferroptosis-based advanced therapies as treatment approaches for metabolic and cardiovascular diseases. Cell Death Differ. (2024) 31:1104–12. doi: 10.1038/s41418-024-01350-1, PMID: 39068204 PMC11369293

