# Supplementary material for: Ferroptosis research based on bibliometric and visual analysis: mechanism exploration and clinical application prospects in gastric cancer, prostate cancer, leukemia, and brain tumors
Source: Front Med (Lausanne). 2025 Oct 24;12:1640497. doi: 10.3389/fmed.2025.1640497 (PMC12592959; doi:10.3389/fmed.2025.1640497)
Supplement: Supplementary file 1 [file Data_Sheet_1.docx]

Supplementary Material: Search Strategy and Data Selection Process

This document provides supplementary information regarding the data collection and screening process for the manuscript titled: "Ferroptosis research based on bibliometric and visual analysis: mechanism exploration and clinical application prospects in gastric cancer, prostate cancer, leukemia, and brain tumors."

This file includes:

The PRISMA 2020 flow diagram illustrating the study selection process.The complete and detailed search strategies used for each of the four cancer types across the three selected databases.

1. Data Selection and PRISMA Flow Diagram

Figure S1. PRISMA 2020 Flow Diagram for Study Selection.

The flowchart below illustrates the systematic process of identifying, screening, and including relevant studies for the bibliometric analysis, following the PRISMA 2020 guidelines. The process began with records identified from three databases (Web of Science, Scopus, and PubMed), followed by rigorous deduplication and eligibility screening, resulting in the final set of articles included in the analysis.


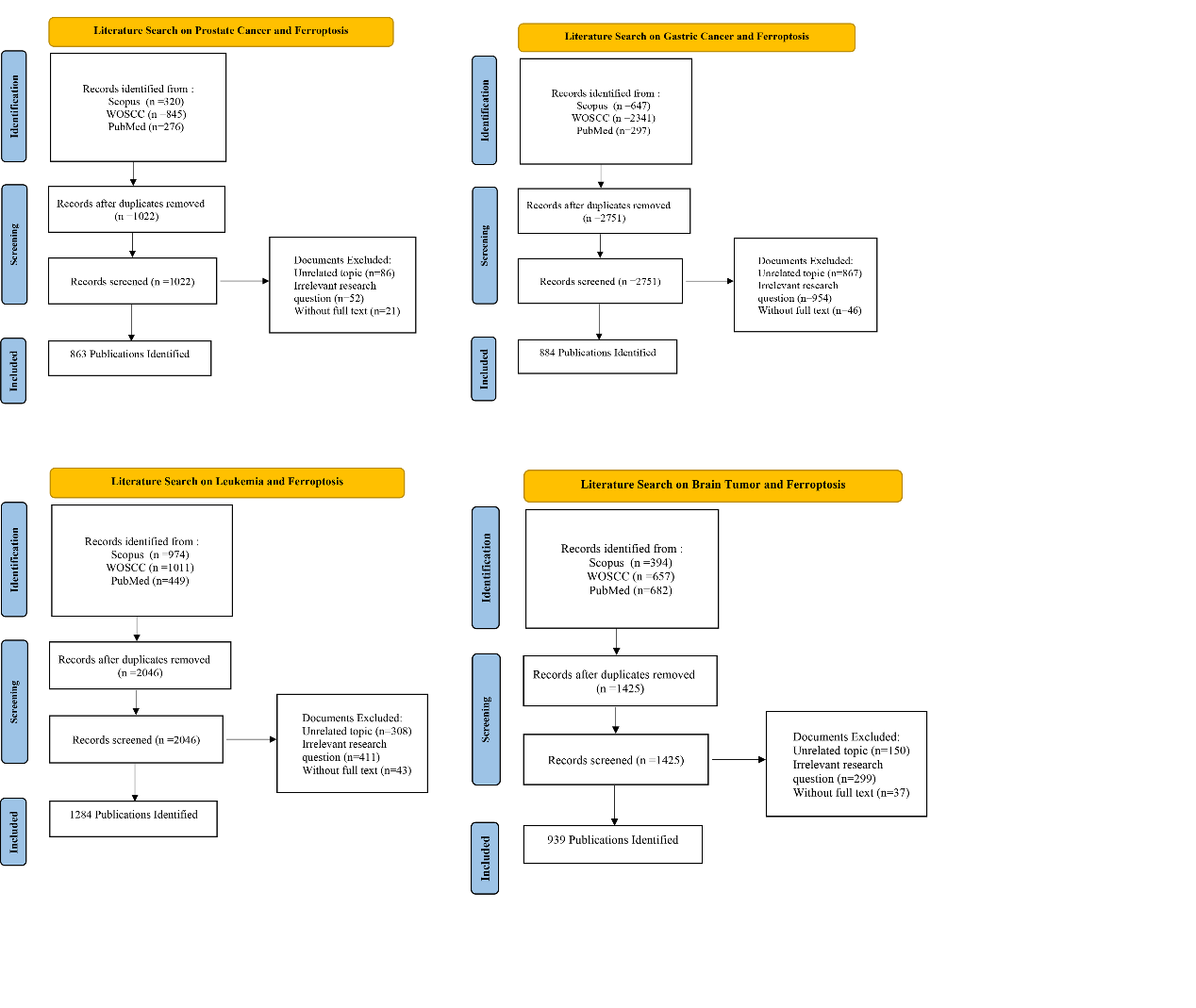


2. Detailed Search Strategies：

Date of last search: January 12, 2025

scopus search strategy
Ferroptosis and prostate cancer,
Filter time: 2015—2025, Filter condition: Article
TITLE-ABS(prostate cancer) OR TITLE-ABS(cancer of the prostate gland) OR TITLE-ABS(prostatic cancer ) OR TITLE-ABS( Prostatic Neoplasms) OR TITLE-ABS(Prostate cancer) AND TITLE-ABS(Ferroptosis ) OR TITLE-ABS( Iron Level) OR TITLE-ABS( iron status ) OR TITLE-ABS( serum iron) OR TITLE-ABS(iron concentration) OR TITLE-ABS(iron metabolism) OR TITLE-ABS(iron homeostasis)

Ferroptosis and gastric cancer
Time limit: 2015-2025, Condition limit: Article
Search strategy:
TITLE-ABS(Ferroptosis ) OR TITLE-ABS( Iron Level) OR TITLE-ABS ( iron status ) OR TITLE-ABS ( serum iron) OR TITLE-ABS (iron concentration) OR TITLE-ABS(iron metabolism) OR TITLE-ABS (iron homeostasis) AND TITLE-ABS(gastric cancer) OR TITLE-ABS(gastric carcinoma) OR TITLE-ABS( cancer of the stomach) OR TITLE-ABS(cancer of stomach) OR TITLE-ABS(Gastric tumor) OR TITLE-ABS(stomach neoplasm)

Ferroptosis and leukemia
Time limit: 2015—2025, Condition limit: Article

TITLE-ABS(Ferroptosis ) OR TITLE-ABS( Iron Level) OR TITLE-ABS( iron status ) OR TITLE-ABS( serum iron) OR TITLE-ABS(iron concentration) OR TITLE-ABS(iron metabolism) OR TITLE-ABS(iron homeostasis) AND TITLE-ABS(Leukemia) OR TITLE-ABS( leucocythemia) OR TITLE-ABS( leucemiz)

Ferroptosis and brain tumor
Time limit: 2015—2025 Condition limit: Article
Search strategy: TITLE-ABS(Ferroptosis ) OR TITLE-ABS( Iron Level) OR TITLE-ABS( iron status ) OR TITLE-ABS( serum iron) OR TITLE-ABS(iron concentration) OR TITLE-ABS(iron metabolism) OR TITLE-ABS(iron homeostasis) AND TITLE-ABS(brain tumor) OR TITLE-ABS(cerebral tumor)

Search in WOSCC database

Ferroptosis and prostate cancer:
Search strategy
((((((TS=(Ferroptosis)) OR TS=(Iron Level)) OR TS=( iron status)) OR TS=(serum iron)) OR TS=(iron concentration)) OR TS=(iron metabolism)) OR TS=(iron homeostasis) AND ((((TS=(prostate cancer)) OR TS=(cancer of the prostate gland)) OR TS=(prostatic cancer)) OR TS=(Prostatic Neoplasms)) OR TS=(Prostate cancer)

Ferroptosis and gastric cancer:
Search strategy
(((((TS=(gastric cancer)) OR TS=(gastric carcinoma)) OR TS=(cancer of the stomach)) OR TS=(cancer of stomach)) OR TS=(Gastric tumor)) OR TS=(stomach neoplasm) AND ((((((TS=(Ferroptosis)) OR TS=( Iron Level)) OR TS=(iron status)) OR TS=(serum iron)) OR TS=(iron concentration)) OR TS=(iron metabolism)) OR TS=(ironV homeostasis)

Ferroptosis and leukemia
Search strategy
((((((TS=(Ferroptosis)) OR TS=( Iron Level)) OR TS=(iron status)) OR TS=(serum iron)) OR TS=(iron concentration)) OR TS=(iron metabolism)) OR TS=(ironV homeostasis) AND ((TS=(Leukemia)) OR TS=(leucocythemia)) OR TS=(leucemiz)
Ferroptosis and brain tumor
Search strategy
(TS=(brain tumor)) OR TS=(cerebral tumor) AND ((((((TS=(Ferroptosis)) OR TS=( Iron Level)) OR TS=(iron status)) OR TS=(serum iron)) OR TS=(iron concentration)) OR TS=(iron metabolism)) OR TS=(ironV homeostasis)

Pubmed

Ferroptosis and prostate cancer
Search strategy
Time limit: 2015--2025
(TS=(prostate cancer) OR (cancer of the prostate gland) OR (prostatic cancer ) OR ( Prostatic Neoplasms) OR (Prostate cancer)) AND (TS=(Ferroptosis ) OR ( Iron Level) OR ( iron status ) OR ( serum iron) OR (iron concentration) OR (iron metabolism) OR (iron homeostasis))

Ferroptosis and gastric cancer

Search strategy:

(TS=(gastric cancer) OR (gastric carcinoma) OR ( cancer of the stomach) OR (cancer of stomach) OR (Gastric tumor) OR (stomach neoplasm)) AND (TS=(Ferroptosis ) OR ( Iron Level) OR ( iron status ) OR ( serum iron) OR (iron concentration) OR (iron metabolism) OR (iron homeostasis))
Time limit: 2015—2025

Ferroptosis and leukemia:
Search strategy:
(TS=(Leukemia) OR ( leucocythemia) OR ( leucemiz)) AND (TS=(Ferroptosis ) OR ( Iron Level) OR ( iron status ) OR ( serum iron) OR (iron concentration) OR (iron metabolism) OR (iron homeostasis))
Time limit: 2015--2025

Ferroptosis and brain tumor:
Search strategy:
(TS=(brain tumor) OR (cerebral tumor)) AND (TS=(Ferroptosis ) OR ( Iron Level) OR ( iron status ) OR ( serum iron) OR (iron concentration) OR (iron metabolism) OR (iron homeostasis))
Time limit: 2015--2025
